# Information Booklet to Enhance Patient Understanding of Services Provided by the Older Adult Mental Health Liaison Team (OA MHLT) at Guy’s & St Thomas’ Hospital: A Quality Improvement Project

**DOI:** 10.1192/bjo.2026.11376

**Published:** 2026-06-30

**Authors:** Mashal Farooq, Tharun Zacharia

**Affiliations:** Guy’s & St. Thomas’ Hospital, London, United Kingdom

## Abstract

**Aims:**

To improve inpatient understanding of liaison psychiatry, specifically the services provided by the Older Adult Mental Health Liaison Team (OA MHLT), by at least 10%, and to improve satisfaction with understanding of key OA MHLT topics among patients. Wehypothesised that a written information booklet would increase patient understanding compared with verbal explanations alone.

Liaison psychiatry is often introduced during acute physical illness, cognitive vulnerability, or heightened distress, which can limit patients’ ability to process and retain verbal information. A written resource is expected to support patient and carer understanding of the rationale for psychiatric involvement, assessment processes and interventions offered.

**Methods:**

OA MHLT staff (n=10) completed a questionnaire to identify high-priority topics for inclusion in a patient information booklet. Inpatients (n=10) were asked how well informed they felt about OA MHLT related topics (including assessment and interventions, psychological support, legal frameworks such as the Mental Health Act and Deprivation of Liberty Safeguards (DoLS), discharge pathways and crisis support) and their satisfaction with their understanding overall (0–5 Likert scale). A patient information booklet was created based on questionnaire findings and distributed to inpatients, after which the patient and staff questionnaire was repeated.

**Results:**

Before booklet distribution, mean staff-rated satisfaction with patient understanding of OA MHLT topics was 1.86/5 and mean patient-rated satisfaction with their understanding was 2.00/5. After booklet distribution, mean staff-rated satisfaction increased to just under 4.6/5 and mean patient-rated satisfaction increased to 4.4/5, exceeding the project aim of ≥10% improvement.

**Conclusion:**

A co-designed OA MHLT patient information booklet was associated with substantially improved staff-rated and patient-rated satisfaction with patient understanding of key liaison psychiatry topics in a small inpatient sample. Further work should evaluatesustainability over time and test the booklet with larger numbers of inpatients. The booklet is due to receive further feedback and final modifications via the service user and carer advisory group.

